# Coevolution of public goods game and networks based on survival of the fittest

**DOI:** 10.1371/journal.pone.0204616

**Published:** 2018-09-25

**Authors:** Guangming Ren, Lan Liu, Mingku Feng, Yingji He

**Affiliations:** 1 School of Optoelectronic Engineering, Guangdong Polytechnic Normal University, Guangzhou, China; 2 School of Electronic & Information, Guangdong Polytechnic Normal University, Guangzhou, China; Centre National de la Recherche Scientifique, FRANCE

## Abstract

We introduce a random strategy update rule for the evolutionary public goods game on networks based on survival of the fittest. A survival cost parameter is introduced to public goods game. Players whose payoffs are below the survival cost will be deleted from the network. The same number of new nodes are randomly connected to the network and randomly designated cooperation or defection. Numerical results show that cooperation can flourish if the multiplication factor of the public goods game is greater than the network degree. We present a simple analytical method to explain this result. The fraction of cooperators reaches the maximum for a suitable survival cost. Furthermore, the initial random network has evolved into a heterogeneous network which facilitates the emergence of the cooperation. Our work could be helpful to understand how natural selection favors cooperation. It suggests a new method to investigate the impact of the survival cost on the evolution of cooperation.

## Introduction

The cooperative phenomena exist universally in nature and human society. Over the past half century, scholars from biology, mathematics, physics, information and even social economy have been investigating the mechanism behind the evolution of cooperation. Cooperators help others at costs to themselves. Defectors can get other people's profit and their short-term gains are higher than cooperators. Why does natural selection favor cooperation? Evolutionary game theory has provided a suitable theoretical framework to study evolution of cooperation among selfish individuals [1–5]. A range of mechanisms have been proposed for the evolution of cooperation, including direct reciprocity, indirect reciprocity, spatial structure, kin selection, group selection, social diversity and voluntary participation[6–8].

In order to understand the cooperation mechanism and promote cooperation, a variety of strategy update rules have been studied, including the preferential selection rule [9], the replication dynamics [10] and Fermi dynamics [11]. For these update rules, individuals must learn strategy information from winners in order to increase their earnings. However, in reality, the strategy information of successful people is not clear, and individuals have their own nature, and they do not always imitate successful people. Another update rule is the Moran process (death-birth update) [12]. For this update rule, in each time step, a randomly selected individual dies and its neighbors compete for the vacancy according to their fitness. The death-birth process can also be understood as the meaning that individuals change strategy and imitate neighborhood strategies according to their fitness. There is also a class of strategy update rules based on aspirations [13–19]. The original strategy is maintained if individual incomes reach the expected value. Otherwise, individuals change the original strategies or learn the neighborhood strategies. The most famous example is the win-stay-lose-shift rule [13]. In addition, Zhang et al. have investigated the influence of survival of the fittest rule on cooperative evolution [20]. They found that cooperation can be enhanced by adding survival of the fittest rule on the basis of replication dynamics.

The strategy update rules based on aspirations can be understood to some extent as survival of the fittest [13–19]. We can think of the expected return as the survival cost, and the strategy change as the death of an individual. However, there are many differences between the two types of rules. First of all, according to the rule based on aspirations, an individual who does not achieve the expected benefit is generally changing his strategy without changing his relationship with other individuals. Second, more importantly, when individuals change their strategies, they either adopt the opposite strategy or learn from their neighbors.

In this work, we introduce a random strategy update rule for the evolutionary public goods game on networks based on survival of the fittest. Individuals whose payoffs are below a set survival cost will die and new individuals are randomly reconnected to the network and randomly assigned cooperation or defection. In our rules, there is no competition, imitation, and individual consciousness. The evolution of cooperation is just a selection process based on the game payoffs. Our model is different from group selection [21–24]. According to group selection, cooperation was costly to individuals, but beneficial to the group, so competition between groups can lead to prosperity of cooperation. As far as we know, our models have not been reported. In literature [20], authors introduced a survival cost parameter, Individuals whose payoffs are below the survival cost will die. However, in their model, new individuals connected to the network according to the preferred rules and learned the strategies of their neighbors, which still is the framework of imitation dynamics. Our work could be helpful to understand how natural selection favors cooperation.

The paper is organized as follows. In Section 2, a public goods game model in an evolutionary network is proposed, where the strategy update rule is designed according to survival of the fittest. Then in Section 3, we have numerically investigated the relationship between the fraction of cooperation and the survival cost. The evolution of the network degree distribution is also analyzed. Finally, we conclude the paper in Section 4.

## The model

Population structure is represented by a graph. The nodes of a graph represent the individuals of an evolutionary game. The edges denote links between individuals in terms of game dynamical interaction. In this work, the game starts in a random network graph. The structure of the graph changes during the evolutionary process.

Each individual and his neighbors form a small group for a public goods game (PGG). In this way, if the individual *x* has *k*_*x*_ neighbors, he will participate in *k*_*x*_ + 1 PGGs that are played in the groups of himself and his *k*_*x*_ neighbors. In each PGG, cooperators with *k*_*x*_ neighbors contribute a cost *c* and the defectors do not contribute. In accordance with previous studies [7], we set *c* = 1/ (*k*_*x*_ + 1). The total contribution is multiplied by a multiplication factor *r* and the result is equally distributed between all *k*_*x*_ + 1 members of the group. Hence, the payoff of an individual *y* with a strategy *s*_*y*_ (1 if C, 0 if D) associated with the PGGs played in a group of an individual *x* is given by

$$P_{y,x}=\frac{r}{k_{x}+1}\sum_{i=0}^{k_{x}}\frac{s_{i}}{k_{x}+1}-\frac{s_{y}}{k_{x}+1}$$ (1)

Where *i* = 0 indicates the individual *x* itself, and *s*_*i*_ is the strategy of individual *i*. If the individual *i* is a cooperator, *s*_*i*_ = 1, if *i* is a defector, *s*_*i*_ = 0. The total payoff of an individual is the sum of payoffs resulted from all related PGGs.

In the end of each generation, all individuals update their strategies synchronously according to survival of the fittest. A parameter *m* is introduced to be the survival cost that permits players participation or not. Each player faces a same survival cost *m*. After each round of playing, players whose total payoffs are less than *m* will be deleted from the network, together with all their links. Conversely, remaining players will survive and continue to play in the next round with unaltered strategies.

When the worse performing players are eliminated, new players will subsequently replace them to maintain a fixed system size *N*. Each newcomer forms *k* connections to preexisting nodes, which are chosen randomly. The parameter *k* is equal to the average degree of the networks. The two strategies, cooperation and defection, are randomly distributed to each newcomer.

## Results

All the simulations are carried out in a population of *N* = 2000 individuals occupying the vertices of the random network graph initially, and the results are robust with the population size. Before the evolution, the two strategies, cooperation and defection, are randomly distributed to all players. So, at the beginning, the fraction of cooperators *f*_c_ of the system is equal to 50%, and the degree of the network is normal distribution.

**Fig 1:**
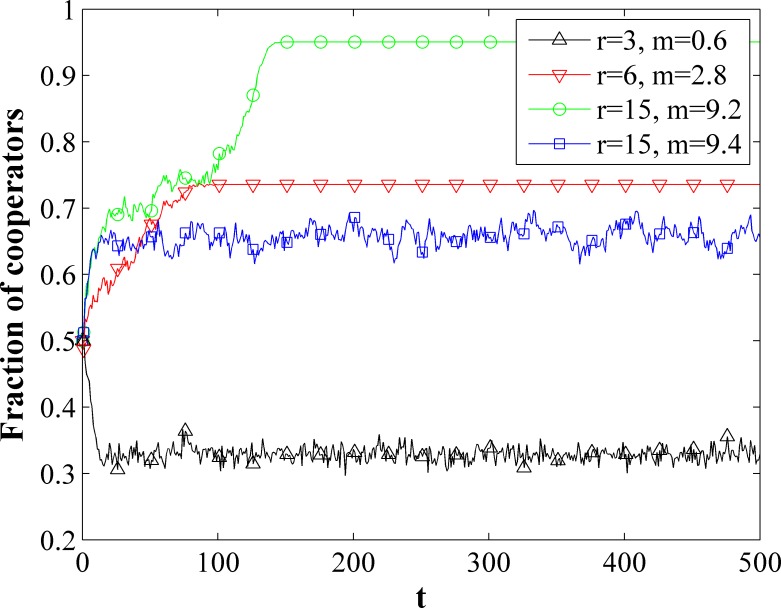
Fraction of cooperators *f*_*c*_ as a function of *t* for different *r and m* on an evolutionary network of average degree *k* = 4.

We analyze relation of the fraction of cooperators *f*_c_ to time step with different multiplication factor *r* and survival cost parameter *m* by using a graph of average degree *k* = 4. The results are showed in the Fig 1. At each time step, according to the above model, the fraction of cooperators *f*_c_ of the system is recorded. When *r* = 3 and *m* = 0.6, the fraction of cooperators *f*_c_ is reduced to less than 50%, that is, the number of defectors in the network is more than that of the cooperators. However, when *r* = 6 and *m* = 2.8, the fraction of cooperators *f*_c_ in the network gradually increased to more than 70%, and gradually reached a stable value with the increase of the simulation time step. As *r* and *m* are further increase to 15 and 9.2 respectively, the steady state fraction of cooperators *f*_c_ increase to over 90%. We should also note that the steady state *f*_c_ will drop significantly to about 60% when *r* = 15 is maintained and *m* is increased a little to 9.4.

In each PGG group, the payoffs of the defectors are always greater than that of the cooperators. Why are more defectors eliminated? In different PGG group, the payoff of the cooperators whose group have more cooperators may be greater than that of the defectors whose group have more defectors. The payoff of all individual is equal to zeros in a network with all defectors. If a cooperator enter this network, his payoff is *rc*/(*k* + 1)—*c* according to our model, the payoff of the defectors in this group is *rc*/(*k* + 1), which is greater than that of cooperator, where *k* is degree of the network. However, the payoff of the defectors in other group is still zero. If *r* > *k*+1, *rc*/(*k* + 1)—*c* > 0. The payoff of the cooperator is greater than most of the defectors. If a suitable *m* is selected, cooperator will flourish.

**Fig 2:**
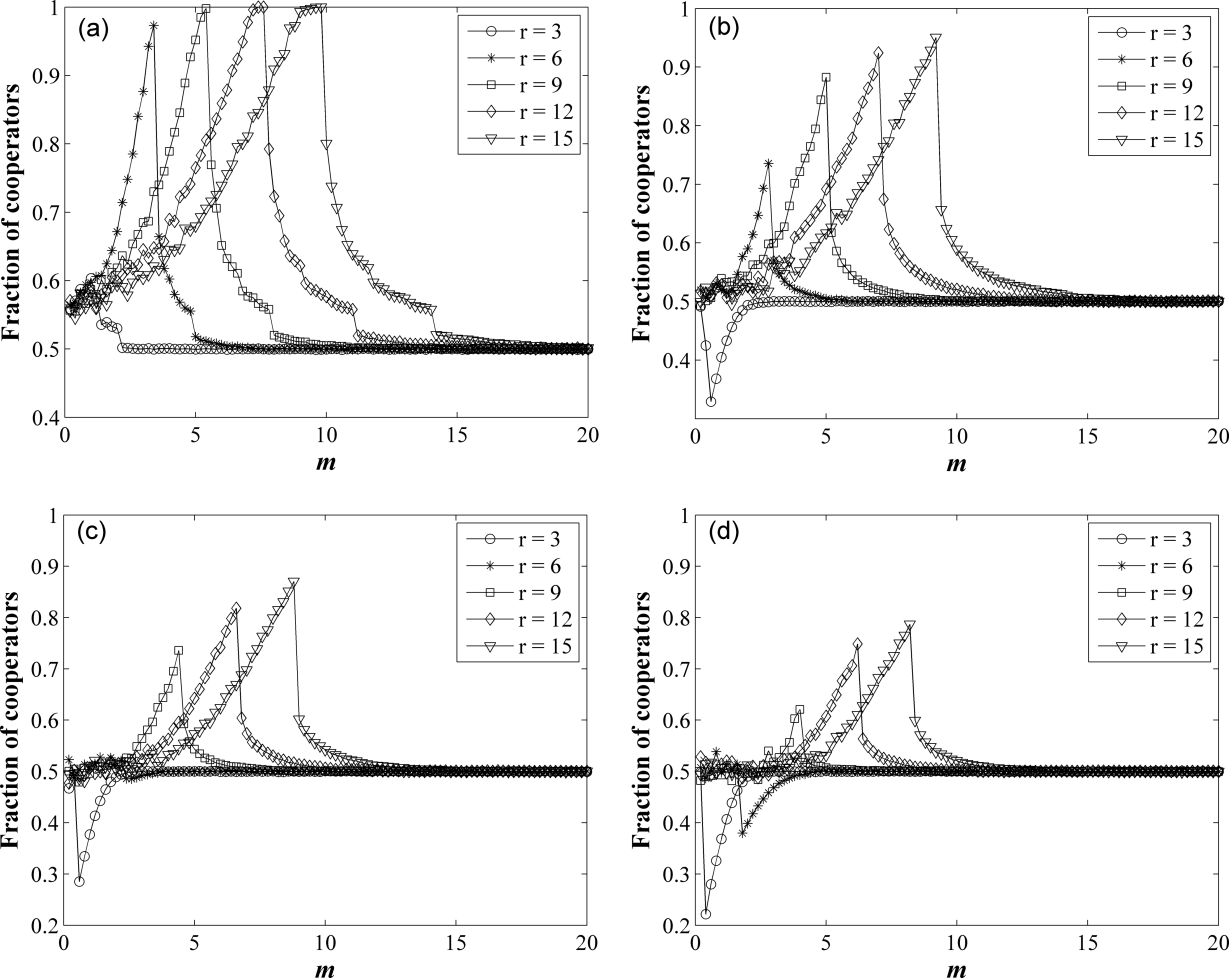
Fraction of cooperators *f*_*c*_ as a function of *m* for different *r* on network of different degree *k*. (a) *k* = 2, (b) *k* = 4, (c) *k* = 6, (d) *k* = 8.

In order to understand these problems, we further analyze the effects of the multiplication factor *r* and the survival cost *m* on the fraction of cooperators *f*_*c*_, as shown in Fig 2. Fraction of cooperators *f*_*c*_ reach a stable value when the time step *t* > 1000. For *t* > 1000, the steady state *f*_c_ is obtained by averaging over the last 1000 generations. By changing the values of *r* and *m*, we get the change curve of the steady state *f*_c_ over *r* and *m*. When *r* > *k*, with increase of *m*, *f*_*c*_ increase from 50% to a max value, then decrease to 50%. When *r* < *k*, with increase of *m*, *f*_*c*_ decrease from 50% to a min value, then increase to 50%. When *r* = *k*, as shown in Fig 2C, *r* = *k* = 6, with increase of *m*, *f*_*c*_ is approximately equal to 50% all along.

When *m* is very small, the payoff of all individuals is larger than *m*. No player is eliminated, so *f*_*c*_ = 50%. With increase of *m*, if *r* > *k*, the average payoff of cooperators is greater than that of defectors, so more defectors are eliminated. Newcomers select randomly cooperate or defect, so *f*_*c*_ >50%. The more increase of *m*, the more obvious this effect is. Because of this effect, we can see that *f*_*c*_ continues to increase to a maximum. When *m* increase to the payoff value of most cooperators, most of the cooperators are also eliminated along with the defectors. And then *f*_*c*_ starts to drop rapidly. When *m* is greater than the payoff of all players, all players are eliminated, so *f*_*c*_ go back to 50%. If *r* < *k*, the average payoff of cooperators is less than that of defectors, more cooperators are eliminated. In this case, *f*_*c*_ decrease from 50% to a min value, then increase to 50%.

Now let's calculate the average payoff of cooperators and defectors in a random network with an average degree of *k*. The probability that each individuals in a network is assigned as a cooperator or defector is 50%. According to our model, on average, the payoff of a cooperator *P*_*c*_ is the sum of the payoffs of his participation in the *k* + 1 game. *P*_*c*_ can be calculated by

$$P_{c}=P_{c}1+P_{c}2+P_{c}3$$ (2)

$$P_{c}1=\frac{(\frac{k}{2}+1)cr}{k+1}-c$$ (3)

$$P_{c}2=\frac{k}{2}(\frac{(\frac{k-1}{2}+2)cr}{k+1}-c)$$ (4)

$$P_{c}3=\frac{k}{2}(\frac{(\frac{k-1}{2}+1)cr}{k+1}-c)$$ (5)

**Fig 3:**
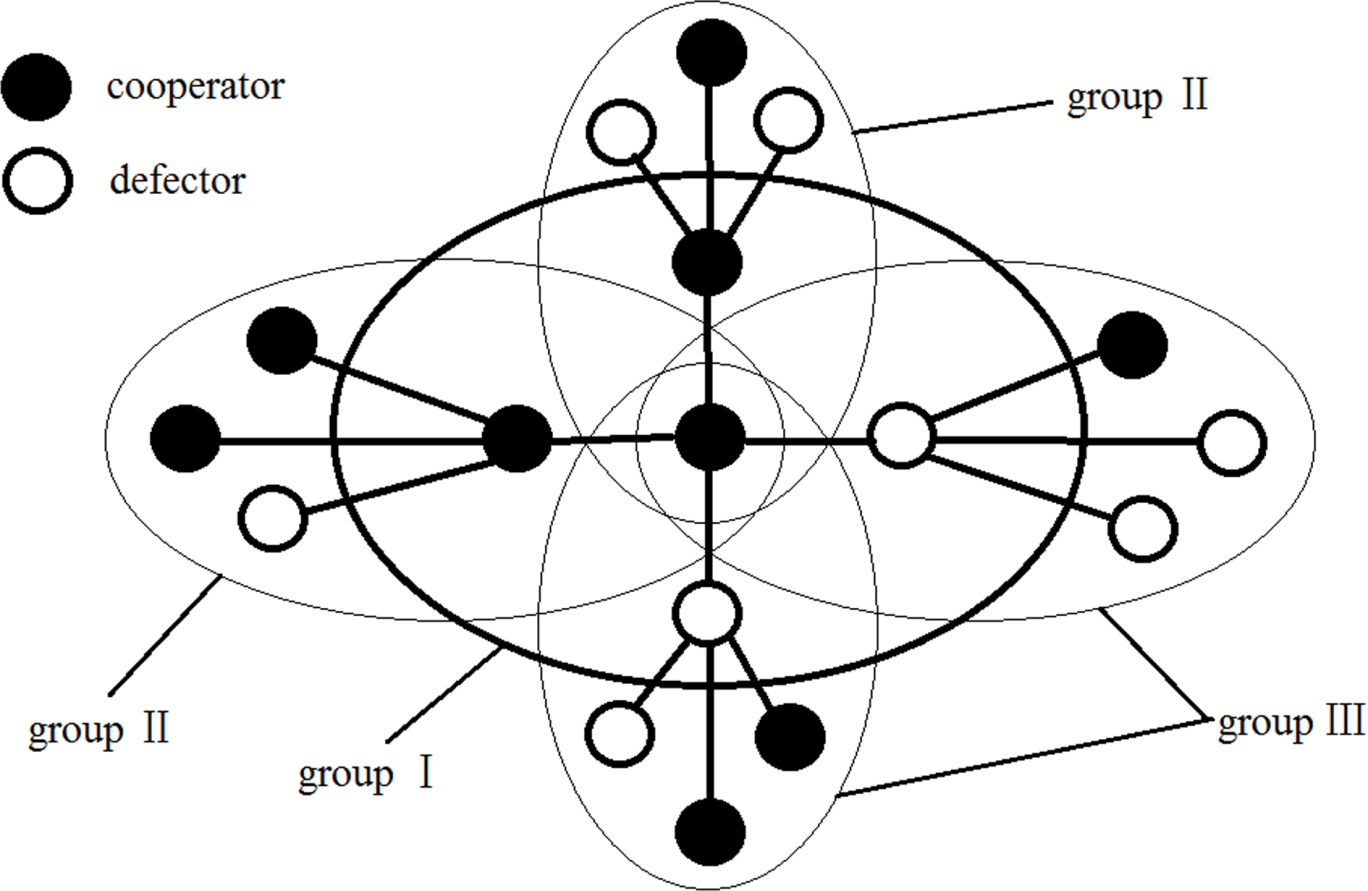
Three types of PGG groups in which a cooperator participate for *k* = 4.

Where *P*_*c*_1, *P*_*c*_2 and *P*_*c*_3 in Eq (2) are payoffs of a cooperator in self-centered PGG group (group Ⅰ), in PGG groups centered around his cooperative neighbors (group Ⅱ) and PGG groups centered around his defective neighbors (group Ⅲ) respectively, as shown in Fig 3. Obviously, there is one group in the first type, *k*/2 groups in the second type, and *k*/2 groups in the third type, all which up to *k* + 1 groups. There are *k* + 1 players in each group. In Eq (3), *k*/2 + 1 represents the number of cooperators in group Ⅰ because 50% of the *k* neighbors are cooperators and plus itself. Two of the *k* + 1 players in group Ⅱ were already cooperators, and the probability of the remaining *k*—1 players being cooperators was 50%, so the number of cooperators in group Ⅱ was (*k*—1)/2 + 2 in Eq (4). In group Ⅲ, there is already a cooperator and a defector, and 50% of the remaining *k*—1 players are cooperators, so the number of cooperators in group Ⅲ was (*k*—1)/2 + 1 in Eq (5).

Similarly, the payoff of a defector *P*_*d*_ is the sum of the payoffs of his participation in the *k* + 1 game. *P*_*d*_ can be calculated by

$$P_{d}=P_{d}1+P_{d}2+P_{d}3$$ (6)

$$P_{d}1=\frac{\frac{k}{2}cr}{k+1}$$ (7)

$$P_{d}2=\frac{k}{2}\frac{(\frac{k-1}{2}+1)cr}{k+1}$$ (8)

$$P_{d}3=\frac{k}{2}\frac{\frac{k-1}{2}cr}{k+1}$$ (9)

From Eqs. (2–9), we can see that the cooperator pays *c* more than the defector in each game. However, the numbers of the cooperators in the group for the defectors are one less than those for the cooperators. By solving the inequality equation *P*_*c*_ > *P*_*d*_, we can get *r* > *k* +1. This result indicates that if *r* > *k* +1, the average payoff of the cooperators is greater than that of defectors, so cooperators can thrive. In the actual numerical simulation, this condition is r > k, which is a little bit less than the theoretical calculation.

**Fig 4:**
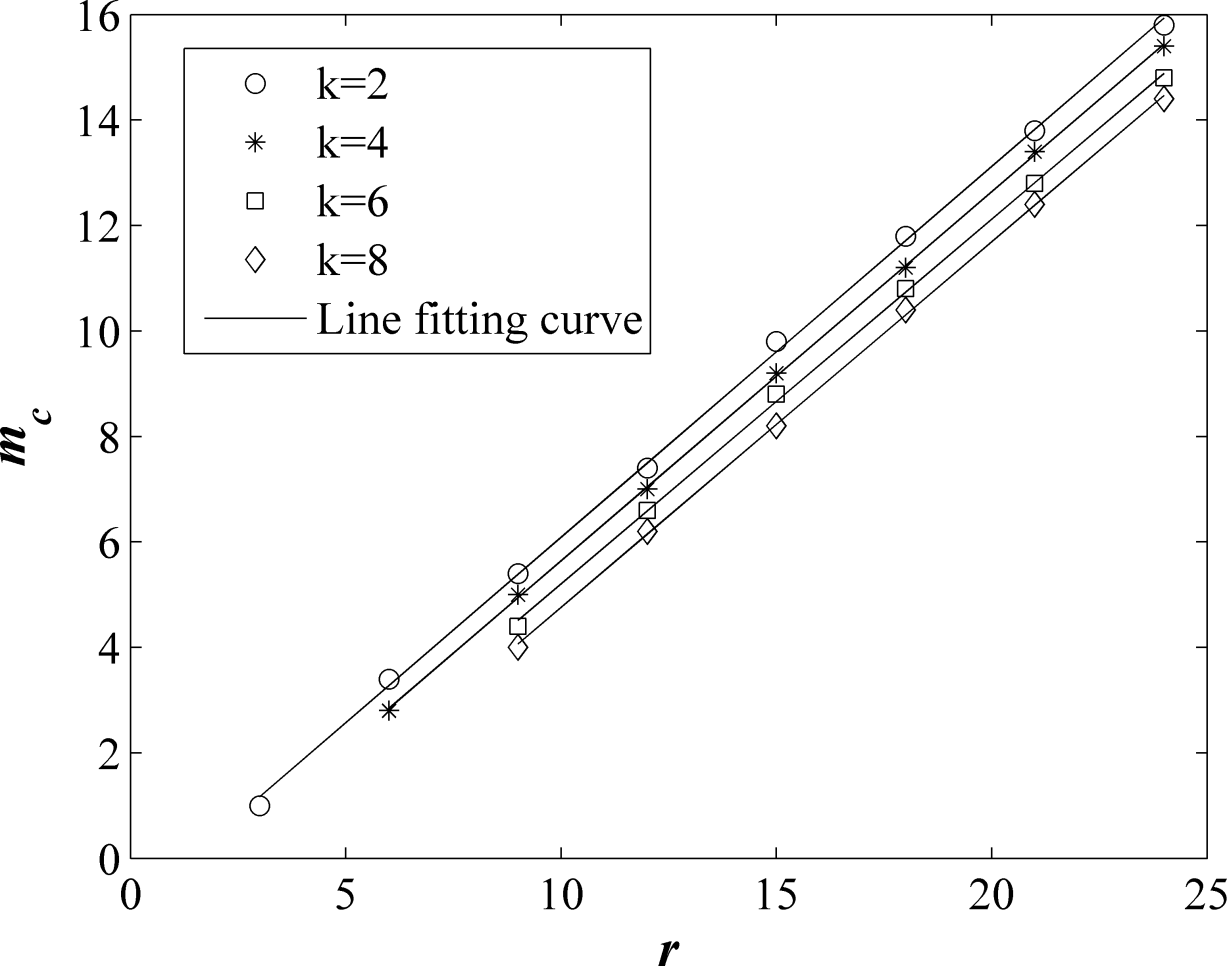
The survival cost *m*_*c*_ as a function of *r* on network of different average degree *k*. Solid lines are line fitting curves.

We should also notice that *m*_c_ increase with increasing *r*, where *m*_c_ is the survival cost *m* when stable-state *f*_*c*_ increase to a maximum. Fig 4 shows *m*_c_ as a function of *r* on network of different average degree *k*. We can see that *m*_c_ and *r* is linear. Solid lines are line fitting curves. The slope of the lines for different *k* is basically the same, about 0.7. For same *r*, the larger the *k*, the smaller the *m*_c_. When *r* < *k*, *f*_*c*_ < 50%. *f*_*c*_ have not a maximum, so the curve in this case isn’t plotted.

When *r* > *k* and *m* < *m*_c_, *f*_*c*_ increases gradually from 50% to a stable constant value with time step *t* increase. After this time step, no individual is eliminated because the payoff of all individuals is higher than *m*. When *r* is unchanged and *m* increases, more individuals are eliminated. If defectors in these eliminated individuals are more than cooperators, *f*_*c*_ will increase and the minimum of the payoff of all individuals *p*_*m*_ also increases. When *p*_*m*_ increases to over new *m*, no player is eliminated, *f*_*c*_ reach stable-state value again. The stable-state *f*_*c*_ increases with growth of *m* until the number of cooperators eliminated is greater than that of the defectors. So *m*_c_ represents the minimum of the payoff of all individuals when stable-state *f*_*c*_ reach maximum. The payoff of the individuals is proportional to *r*, so *m*_c_ is proportional to *r*.

**Fig 5:**
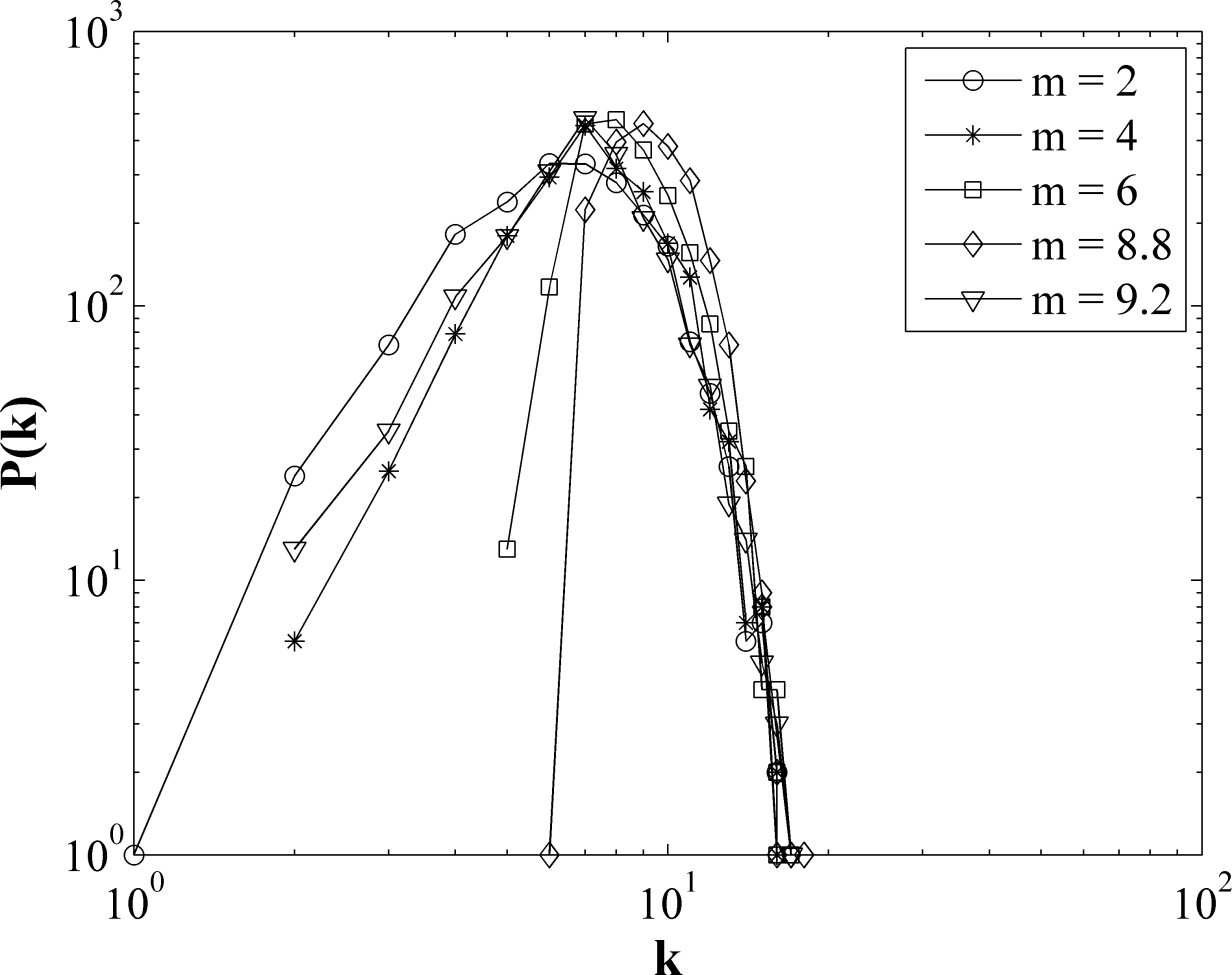
The networks degree distributions with different *m* values for *r* = 15 on original network of average degree *k* = 6.

Finally, we focus our attention on the evolving network structure, e.g. the resulting degree distributions, when the system reaches a steady state. Fig 5 Shows the networks degree distributions with different *m* values for *r* = 15 on network of *k* = 6. When *m* is small, payoff of all players is higher than *m*. No player is eliminated. The degree of network is approximately normal distribution. With the increase of *m*, the small degree nodes are eliminated due to the low payoff. So the network structure changes, the nodes with large degree increase, and the small degree nodes gradually disappear. When *m* reaches *m*_*c*_ = 8.8, this effect reaches the maximum. If *m* goes up further, many large degree nodes are also eliminated and small degree nodes appear in the network again.

## Discussion and conclusions

In this paper we have studied the coevolution of cooperation and network based on survival of the fittest. In our model, individuals who earn less than the survival cost are eliminated and new individuals randomly choose cooperation or defection. The numerous simulations suggest that cooperation can emerge and thrive if the multiplication factor of public goods game *r* is greater than the network average degree *k*. This conclusion is similar to previous studies shown in Refs. [6,7,12] with different models, In reference [12], authors described a simple rule: natural selection favors cooperation, if the benefit of the altruistic act, *b*, divided by the cost, *c*, exceeds the average number of neighbors, *k*, which means *b*/*c* > *k*. In our model, the benefit of the altruistic act *b* = *rc*, so our parametric condition *r* > *k* is consistent with previous studies. However, our model is quite different from previous work. First of all, the updating rules adopted in reference [12] mainly include death–birth updating, birth–death updating and imitation updating, which are essentially imitative dynamics. In our model, the individual does not actively update the strategy. The updating of the strategy originates from an elimination process, that is, the payoff of the individuals below the cost of survival are eliminated and the new individuals are randomly assigned to the strategy, which we call elimination dynamics. In addition, the previous work mainly analyzed the weak selection situation. The fitness of a player is given by 1 –*w* + *wP*, where *P* is the payoff of players and *w* measures the intensity of selection. Strong selection means *w* = 1. Weak selection means *w* ≪ 1. In our model, the fitness of the player is equal to his payoff, that is, *w* = 1, which is the strong selection situation.

Obtaining analytical insights into our results seems to be a natural topic. In reference [12], authors have calculated the fixation probability of a randomly placed mutant for any two-person, two-strategy game on a regular graph by using pair approximation and diffusion approximation. The fixation probability is the probability that a single cooperator starting in a random position turns the whole population from defection to cooperation. If selection neither favors nor opposes cooperation, then this probability is 1/*N*, which is the fixation probability of a neutral mutant, where *N* is the population size. If the fixation probability of a single cooperator is greater than 1/*N*, then selection favors the emergence of cooperation. They have found that cooperators have a fixation probability greater than 1/*N* if *b*/*c* > *k*. In our model, selection favors the emergence of cooperation on the condition that the average payoffs of the cooperators is higher than those of the defectors. After a simple calculation, we get the result *r* > *k* + 1. The core idea of our calculation method is that if we only consider the payoff generated by the individual's own cost, when *r* > *k* + 1, the payoff of a cooperator is *rc*/(*k* + 1), which is greater than his cost *c*, and the defector does not pay, there is no benefit. The calculation process is still not perfect, but we provide a new method to solve such problems.

Another important result of this work is that the survival cost has a great influence on the evolution of cooperation. Too big or too small survival cost is not conducive to the flourish of cooperation, and the cooperation ratio reaches the maximum when the survival cost is equal to a suitable value *m*_c_, which increase linearly with multiplication factor *r*. These results are similar to previous studies based on aspiration update [13–19]. In reference [14], Chen et al. had studied the evolutionary prisoner’s dilemma game on small-world networks for different average aspiration levels based on aspiration update rule. According to the aspiration update rule, during the evolutionary process, player *x* will compare the collecting payoff *P*_*x*_ from neighbors with the aspiration level *P*_*xa*_, and change its current strategy to its opposite strategy with a probability depending on the difference (*P*_*x*_−*P*_*xa*_). They found that there exists an appropriate intermediate aspiration level leading to the maximum value of cooperation. Since then, the role of aspirations in evolutionary games had received a lot of attention. In reference [16], authors incorporate individual aspirations into the traditional imitation rule, and investigate how cooperation evolves under the aspiration-based conditional learning in the spatial prisoner’s dilemma game. Although these studies produced similar results to ours, our model is substantially different from previous work. In our model, more intuitive results can be obtained by introducing the concept of the survival cost.

In recent years, coevolutionary games on networks have received great attention. Scholars try to study the influence of evolutionary game dynamics on networks, and explain the emergence mechanism of complex network structures [25–29]. In our model, the degree distribution of the network changes gradually with the increase of time step. The initial random network has been evolved into a heterogeneous network which facilitates the emergence of the cooperation. Evolution of network structure may present an exciting challenge for future research.

## Supporting information

S1 Appendix Computer program for the paper. (ZIP)
