# Supplementary material for: Coevolution of public goods game and networks based on survival of the fittest
Source: PLoS One. 2018 Sep 25;13(9):e0204616. doi: 10.1371/journal.pone.0204616 (PMC6155537; doi:10.1371/journal.pone.0204616)
Supplement: S1 Appendix — (ZIP) [file pone.0204616.s001.zip › readme.docx]

Run Net1_2.m to generate network data files.

Run Game1_2d.m to generate numerical results files.

Run figure1draw.m to generate figure files.

And so on.
